# Reliability and Anatomical Agreement of High-Resolution Ultrasound for Measuring the Length and Thickness of the A1 Pulley: A Cadaveric Study

**DOI:** 10.3390/life16060867

**Published:** 2026-05-22

**Authors:** Xeber Iruretagoiena, Marc Blasi, Ramón Balius, Xavier Sala, María Garralda, Javier De la Fuente

**Affiliations:** 1 Deusto Physical TherapIker, Physical Therapy Department, Faculty of Health Sciences, University of Deusto, 20012 San Sebastián, Spain; xeber.iruretagoiena@deusto.es; 2 Eskura Osasun Zentroa, 20200 Beasain, Spain; 3 Sputnik Investigación, 28232 Madrid, Spain; 4 Department of Plastic Surgery, Hospital Germans Trias I Pujol, 08916 Barcelona, Spain; marcblasibrugue@gmail.com; 5 Consell Catala de l’Esport, Generalitat de Catalunya, 08950 Barcelona, Spain; ramonbaliusmatas@gmail.com; 6 Sport Medicine and Imaging Department, Clínica Diagonal, 08950 Barcelona, Spain; 7 Anatomy and Embryology Department, School of Medicine, Universitat de Barcelona, 08007 Barcelona, Spain; xavi.sala.blanch@gmail.com; 8 Department of Anesthesiology, Hospital Clínic de Barcelona, 08036 Barcelona, Spain; 9 Department of Orthopedics, University Hospital of Navarra, 31008 Pamplona, Spain; m96garralda@gmail.com; 10 Department of Orthopedics, Clínica Pakea-Mutualía, 20018 San Sebastián, Spain

**Keywords:** high-resolution ultrasound, A1 pulley, trigger finger, cadaveric study, reliability and anatomical agreement

## Abstract

Accurate assessment of the A1 pulley is essential for the diagnosis and treatment of trigger finger, particularly in ultrasound-guided percutaneous release. Although high-resolution ultrasound is widely used to evaluate pulley morphology, the validity of sonographic measurements of A1 pulley length has not been clearly established against anatomical reference standard. This study evaluated the reliability and validity of ultrasound for measuring A1 pulley length and thickness in human cadavers and assessed the reproducibility of A2 pulley length. Twenty fingers from five fresh-frozen cadaveric hands were examined. Two blinded expert musculoskeletal sonographers independently performed ultrasound acquisition and measurements of A1 and A2 pulley length and A1 pulley thickness, while a third blinded observer obtained anatomical measurements after meticulous dissection using a digital caliper. Ultrasound systematically overestimated A1 pulley length compared with anatomical measurements and showed very poor reliability (ICC = 0.05) with wide limits of agreement. In contrast, A2 pulley length showed high interobserver reliability (ICC = 0.83) and relatively better agreement with anatomical values, whereas A1 pulley thickness showed moderate reproducibility (ICC = 0.61). Overall, A1 length measurements showed substantial variability and limited agreement, while A2 length and A1 thickness appeared more consistent within this experimental setting. These findings should be interpreted within the limitations of a cadaveric model.

## 1. Introduction

Stenosing flexor tenosynovitis, commonly referred to as trigger finger, is one of the most frequent hand disorders encountered in clinical practice and is primarily associated with pathological changes in the A1 pulley [1]. The A1 pulley plays a key role in regulating flexor tendon gliding at the metacarpophalangeal joint, and its fibrocartilaginous thickening represents the hallmark structural abnormality in symptomatic cases [1]. Accurate assessment of A1 morphology is therefore clinically relevant for both diagnosis and treatment.

Despite its apparent simplicity, the A1 pulley exhibits considerable anatomical variability. Its proximal fibers may blend with the volar plate of the metacarpophalangeal joint, while its distal fibers often show a gradual and indistinct transition toward the A2 pulley [1]. This lack of sharply defined anatomical borders complicates both anatomical characterization and imaging-based identification, particularly when attempting to define the true longitudinal extent of the A1 pulley [2]. This reflects not only macroscopic variability but also the microstructural continuity of the retinacular system [3,4].

High-resolution ultrasound is widely used for the evaluation of the flexor tendon sheath and has become an essential tool in the diagnosis and ultrasound-guided treatment of trigger finger [5]. Sonography allows dynamic assessment and visualization of pulley thickening; however, the thin structure of the A1 pulley, its oblique fiber orientation, and its variable transition to A2 make precise boundary identification challenging [6]. Although ultrasound-derived measurements of A1 pulley length are frequently reported, their reliability and anatomical validity have not been rigorously established. This represents a critical gap, as these measurements are often implicitly used as anatomical references in both diagnostic evaluation and ultrasound-guided interventions, despite the lack of evidence supporting their reproducibility.

The primary objective of this cadaveric study was to evaluate the reliability and validity of high-resolution ultrasound for measuring the anatomical length of the A1 pulley using direct anatomical dissection as the reference standard. Secondary objectives were to assess the reproducibility of ultrasound measurements of A1 pulley thickness and A2 pulley length.

## 2. Materials and Methods

Using fresh human cadaver specimens and a cross-sectional blinded, multi-observer design, ultrasound measurements obtained by two experienced sonographers were compared directly with caliper measurements following meticulous anatomical dissection.

This cross-sectional cadaveric study was conducted using fresh-frozen human hands to evaluate the reliability and validity of high-resolution ultrasound to identify the anatomical borders and measure the true length of the A1 pulley. A total of 20 fingers (index, middle, ring, and little fingers) from five fresh-frozen cadaveric hands were included. Digits with prior surgery, scars, deformities, advanced osteoarthritis, or visible tendon sheath pathology were excluded. Thumbs were also excluded due to their distinct anatomical configuration and pulley system, which differs from that of the long fingers. All specimens were obtained from bodies donated to the Faculty of Medicine and Health Sciences (Clinic Campus) at the University of Barcelona. Institutional review board approval was obtained prior to the study. Donor identities were anonymized, and hands were thawed at room temperature for 24 h prior to assessment to replicate physiological tissue elasticity. Although no formal a priori sample size calculation was performed due to the exploratory cadaveric nature of the study, the number of specimens included was comparable to or exceeded that used in previous validated anatomical and ultrasound reliability studies of the digital flexor pulley system.

Ultrasound examinations were performed using a high-resolution Canon Aplio a US machine equipped with both linear and high-frequency hockey-stick (i22LH8) transducers, allowing detailed visualization of the flexor tendon sheath (Canon Medical System^®^, Tustin, CA, USA). The hockey-stick transducer was preferentially used for the assessment of A1 pulley length and thickness due to its higher spatial resolution for superficial structures, whereas the linear transducer was used for A2 pulley length measurements. All scans were conducted with abundant gel to minimize transducer pressure and prevent distortion of the thin fibrocartilaginous pulley structures. Hands were positioned supine with the wrist in neutral and digits fully extended. This static posture aimed to reduce tendon tension and optimize depiction of the retinacular system, as recommended in prior imaging studies of digital pulleys [5].

**Figure 1:**
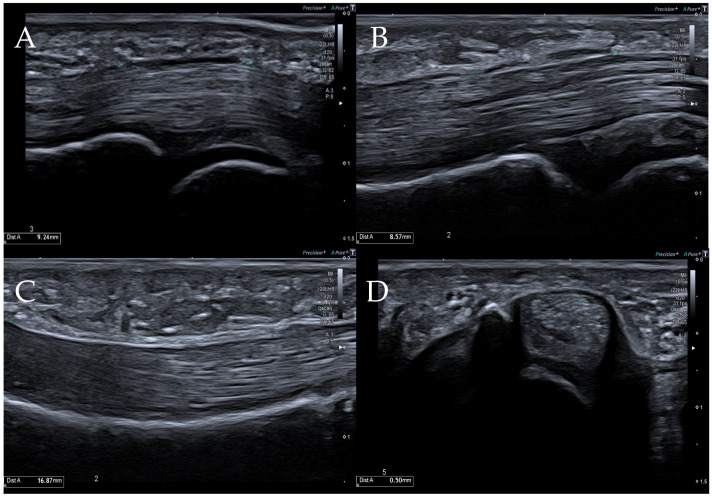
Long-axis ultrasound measurement of the A1 pulley (**A**,**B**) and A2 pulley (**C**) length, with the distal aspects oriented to the left and the proximal aspect to the right. Short-axis image (**D**) illustrates ultrasound measurement of the A1 pulley thickness, with the ulnar side shown on the left and the radial side on the right.

Two experienced musculoskeletal sonographers (O1 and O2), with over 30 and 8 years of dedicated hand ultrasound experience, respectively, independently performed all examinations. Both were blinded to each other’s measurements and specimen order. Ultrasound assessment included: A1 and A2 pulley length (mm) in the long axis relative to the flexor tendon and maximum A1 pulley thickness (mm), measured at its point of greatest thickness in the short axis (Figure 1). Each measurement was performed three times using the device inbuilt caliper, and the mean value was used for analysis. All images were stored with measurement annotations for subsequent analysis. Each observer independently performed both ultrasound image acquisition and measurement for all specimens, without access to pre-recorded or shared images, ensuring that reliability assessment reflected both acquisition and measurement variability.

To determine pulley limits common criteria were used by both observers. For A1 proximal border, visualization of the fibrocartilaginous thickening arising from the volar plate and metacarpal head was sought, whereas the distal A1 border was determined at the point where pulley thickening abruptly changed before the origin of the A2 pulley. A2 borders were defined using classical retinacular landmarks [5,7]. In addition, prior to static measurements, passive flexion–extension of the digits was performed to facilitate identification of the pulley system by differentiating the moving flexor tendon from the relatively fixed pulley structures, particularly in the long-axis view. Subtle adjustments of the insonation angle were also performed, using anisotropy as an additional aid to better delineate pulley tissue from adjacent structures.

**Figure 2:**
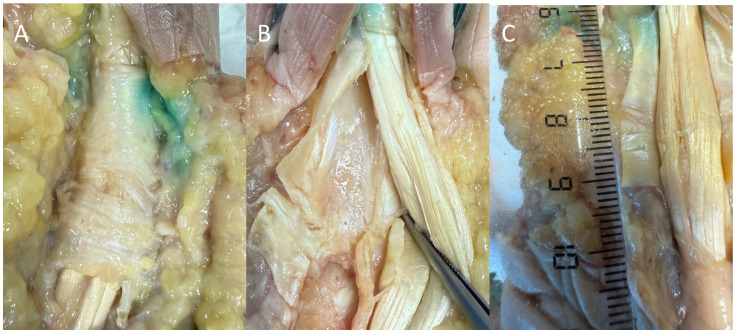
Cadaveric image of the A1 and A2 pulleys. (**A**) Intact flexor pulley system. (**B**) Sectioned A1 and A2 pulleys exposing underlying structures. (**C**) Direct measurement of A1 and A2 pulley length using a digital caliper.

Following ultrasound assessment, each finger was dissected by a third independent examiner (O3) blinded to sonographic measurements. Stratigraphical dissection was performed under loupe magnification to expose the flexor tendon sheath (Figure 2). The dissection strategy followed methodologies used in prior validated anatomical pulley studies [8]. A precision digital caliper (0.01 mm accuracy) was used to measure A1 and A2 pulley length (Qfun^®^ digital caliper, Hong Kong, China, 0–150 mm, CN). Measurements were taken at the midline of the pulley to minimize curvature-related errors. Measurement of A1 pulley thickness was not performed using the caliper due to the limited accuracy of direct thickness assessment in such thin fibrocartilaginous structures.

A block randomization procedure (ABC/BAC) was used to determine the order of evaluations, with A = O1, B = O2, and C = O3. Ultrasound measurements were always performed before anatomical dissection. All observers remained blind throughout the entire process.

Descriptive data were expressed as mean, standard deviation, and range. Inter-observer reliability between O1 and O2 was assessed using the Intraclass Correlation Coefficient (ICC) based on a two-way random-effects model with absolute agreement, calculated on the mean of three repeated measurements per observer, as well as Lin’s concordance correlation coefficient, and Bland–Altman plots. ICC values were interpreted according to commonly used thresholds: <0.5 poor, 0.5–0.75 moderate, 0.75–0.9 good, and >0.9 excellent reliability. Validity was evaluated by comparing O1 and O2 measurements against the anatomical reference values obtained by O3 using Passing–Bablok regression and the CUSUM test for linearity, as well as Bland–Altman analysis. A significance threshold of *p* < 0.05 was used for all analyses. Statistical procedures were conducted using R.

## 3. Results

A total of 20 fingers were measured by three observers (O1, O2 and O3). No pathological findings were identified in any of the specimens; therefore, no fingers were excluded from the analysis.

### 3.1. Descriptive Measurements

Ultrasound measurements tended to overestimate A1 pulley length when compared with anatomical caliper measurements, with greater overestimation observed in observer 1 than in observer 2. In contrast, A2 pulley length measurements were broadly similar between ultrasound observers and showed closer agreement with anatomical reference values. Measurements of A1 pulley thickness were consistent between observers, with only minimal differences (Table 1).

### 3.2. Interobserver Reliability

Interobserver reliability for A1 pulley length was very poor, indicating substantial variability between ultrasound observers and limited reproducibility. In contrast, A2 pulley length demonstrated high interobserver reliability, while A1 pulley thickness showed moderate agreement between observers. Overall, reliability was highest for A2 length, intermediate for A1 thickness, and lowest for A1 length. Confidence intervals for ICC values are provided in Table 2, together with Lin’s concordance correlation coefficients.

### 3.3. Validity

Validity analyses demonstrated a systematic overestimation of A1 pulley length by ultrasound, with wide limits of agreement relative to anatomical measurements. This pattern was consistent across observers, indicating limited validity for ultrasound-based A1 length assessment. In contrast, A2 pulley length showed smaller bias and better agreement with anatomical reference values, suggesting a more accurate sonographic estimation of A2 length compared to A1. Passing–Bablok regression indicated the presence of both proportional and constant differences between ultrasound and anatomical measurements for A1 length. Corresponding confidence intervals for intercept and slope are reported in Table 3. The CUSUM test for linearity did not show significant deviation from linearity for all comparisons (*p* > 0.05).

## 4. Discussion

This cadaveric study evaluated the reliability and validity of high-resolution ultrasound for measuring the anatomical length and thickness of the A1 pulley, using meticulous anatomical dissection as the reference standard. The main finding is a clear dissociation between sonographic behavior of the A1 and A2 pulleys. A2 length showed good inter-observer reliability and acceptable agreement with anatomical measurements. In contrast, A1 length demonstrated poor reproducibility, limited agreement with the anatomical reference, and systematic overestimation by ultrasound, indicating that this parameter cannot be considered sufficiently consistent for reliable clinical use. In contrast, A1 pulley thickness showed moderate inter-observer reliability and remained a relatively consistent, clinically relevant and reproducible parameter.

The difficulty in reliably measuring A1 length is strongly rooted in its intrinsic anatomical variability [9]. Classical anatomical descriptions have consistently highlighted that the A1 pulley is a short annular structure with poorly defined borders, particularly distally, where its fibers may partially overlap with or gradually transition into the proximal portion of the A2 pulley [3]. Histological studies have further demonstrated that the fibrocartilaginous composition of A1 is thinner, more irregular, and less homogeneous than that of A2, resulting in a structural continuum rather than a sharply demarcated boundary between the two pulleys [4]. This reflects the underlying microstructural continuity of the retinacular system and helps explain why attempts to define a single, reproducible longitudinal extent of A1 are prone to error [3].

In line with this intrinsic anatomical variability, the present results also help contextualize the wide range of A1 pulley lengths reported in prior cadaveric studies. Reported values ranging approximately from 6 to 10 mm appear to be strongly influenced by how proximal and distal limits are defined, rather than reflecting true anatomical consistency [1,9,10,11]. In our dissections, the most reproducible anatomical reference for the proximal origin of A1 was located around the mid-portion of the metacarpal head, rather than its distal edge or surface landmarks. This observation is particularly relevant when contrasted with previous work relying on cutaneous or palpatory landmarks, such as that described by Rojo et al., which may systematically incorporate adjacent retinacular or volar plate structures into the measured A1 length [12,13,14]. Although this reference point is not directly sonographic, its anatomical consistency supports its relevance for interpreting both cadaveric and imaging-based measurements and should be considered when defining anatomical standards.

From an ultrasound perspective, the systematic overestimation of A1 length observed in both observers reflects the challenge of differentiating the pulley from adjacent structures. Proximally, discrimination between A1 fibers and the synovial sheath remains difficult because both structures are thin and differ mainly in thickness rather than echogenicity, a feature that remains difficult to assess reliably even when using optimized acquisition strategies, including the use of a thick gel stand-off technique to avoid direct transducer-skin contact and systematic modulation of the insonation angle to exploit anisotropy [15]. The distal border introduces a different difficulty, largely influenced by anatomical variation: the A1 pulley may be continuous with A2, separated from it, or interrupted by cruciform fibers, making this transition hard to interpret sonographically and sometimes even during dissection [9]. Additionally, the A1 pulley may consist of one or multiple fibrous rings, whose intermittent thickness further complicates the definition of its true borders [16]. These factors indicate that, in a subset of cases characterized by anatomical variability, defining a single distal “endpoint” of the A1 pulley may not be feasible in a reliable manner, either sonographically or during anatomical dissection, even when optimal imaging techniques are applied. Even under direct visualization, the definition of these anatomical borders remains partially observer-dependent. These limitations were consistently reflected in Bland–Altman and Passing–Bablok analyses, which demonstrated positive bias and wide limits of agreement relative to anatomical measurements.

Despite these limitations, ultrasound remains highly valuable for evaluating A1 pulley pathology. Multiple sonographic studies have shown that pathological thickening, loss of the normal hyperechoic profile, and dynamic tendon notching during flexion are robust imaging features of stenosing flexor tenosynovitis [17]. Histopathological investigations further demonstrate that fibrocartilage hypertrophy, collagen disorganization, and fibrovascular proliferation predominantly affect the mid-to-distal portion of the A1 pulley, directly correlating with mechanical stenosis and impaired tendon gliding. In our study, A1 pulley thickness demonstrated moderate inter-observer agreement and only minimal systematic differences between observers, supporting its role as a more dependable sonographic marker than A1 length for diagnosis, monitoring, and treatment planning [18]. This interpretation is consistent with previous ultrasound studies of trigger finger, in which focal A1 thickening emerges as the most reproducible and clinically meaningful imaging parameter, closely related to symptom severity and flexion contracture [19]. In healthy volunteers, high-resolution ultrasound has further demonstrated that A1 thickness can be measured reproducibly and exhibits predictable variation with demographic factors, reinforcing its value as a quantitative parameter in both research and clinical settings [20].

It is important to emphasize that the present findings are derived from a cadaveric model using non-pathological specimens and therefore do not directly reflect clinical diagnostic performance or procedural outcomes. Accordingly, the following clinical considerations should be interpreted as hypothesis-generating rather than definitive. These findings have potential implications for ultrasound-guided trigger finger release, as they suggest that current imaging-based assumptions regarding A1 length may not provide a sufficiently reliable basis for procedural guidance. While precise knowledge of A1 longitudinal extent may not be strictly required to achieve symptomatic relief, cadaveric studies have shown that procedural safety depends on avoiding excessive extension toward the A2 pulley and protecting adjacent structures [2,20]. Our data suggest that ultrasound is well suited to guide instrument placement, identify focal thickening, and monitor real-time tendon–pulley interaction, but should not be relied upon to define strict longitudinal limits of A1. Systematic overestimation of A1 length may inadvertently promote overly extensive releases, whereas difficulty identifying the A1–A2 transition may introduce uncertainty during decision-making. A clinically oriented approach that prioritizes thickness-based assessment, functional response, and anatomical awareness of the proximal A2 landmark appears more appropriate.

In contrast, A2 length exhibited higher inter-observer reliability and better agreement with the anatomical measurements compared to A1. This is consistent with previous cadaveric and clinical research showing that A2 is the longest and most consistent annular pulley, with clearer cortical anchors and more reproducible ultrasound landmarks than other pulleys [21]. Prior research on ultrasound diagnosis of A2 and A4 pulley tears, as well as on tendon-to-bone distance in partial and complete A2 ruptures, has further demonstrated the ability of high-resolution ultrasound to detect subtle structural changes at this level with good diagnostic accuracy [22]. In this context, the proximal border of the A2 pulley may present a relatively stable and reproducible sonographic landmark when compared with the variable distal extent of A1. Accordingly, our findings suggest a relative advantage of A2-related measurements over A1 for imaging-based assessment of the flexor pulley system; however, these results should be interpreted with caution given the limited sample size and cadaveric study design and should not be considered definitive.

The discrepancies between ultrasound and anatomical measurements in our study can be explained by both anatomical and technical factors. The thinness of A1, its fiber discontinuity, anatomical variations, and its close integration with the volar plate and the proximal fibers of A2 create an intrinsically complex structure for ultrasound to resolve [6,15,16]. In addition, subtle changes in tendon tension, probe pressure, and anisotropy may obscure the distal hyperechoic border of the pulley or blend it into the adjacent sheath. Similar sources of variability have been highlighted in the literature on tendon-to-bone distance and ultrasound diagnostic criteria for pulley injuries, where heterogeneity of technique (landmarks, finger position, loading) leads to inconsistent cut-off values and variable agreement between studies [21,23]. These considerations help explain not only the broad range of A1 lengths reported in anatomical and imaging studies, but also the positive bias and wide limits of agreement observed here.

This study has several strengths, including the use of fresh cadaveric specimens to preserve soft-tissue architecture, blinded multi-observer assessment, and application of robust reliability and validity statistics. Nevertheless, some limitations must be acknowledged. First, all examined pulleys were non-pathological, which may have influenced sonographic recognition, as pathological A1 pulleys are typically thickened and may present clearer sonographic delineation than physiological pulleys in cadaveric specimens. Therefore, the difficulty in identifying A1 borders observed in this study may represent a worst-case anatomical scenario and may differ from clinical settings involving stenosing tenosynovitis. Even under direct visualization, defining the exact borders of A1 remains challenging, meaning that caliper measurements, although the best available anatomical standard, are not entirely free of uncertainty. Second, although each observer repeated measurements three times to reduce random error, formal intra-observer reliability was not assessed, as only averaged values were retained for analysis, preventing calculation of intra-observer ICCs. This represents a limitation of the study. Third, digit-specific analyses (index, middle, ring, and little fingers) were not performed due to the limited sample size per digit, which would have resulted in underpowered comparisons and potentially unreliable subgroup interpretations. In addition, fingers were analyzed as independent observations despite being obtained from a limited number of cadaveric hands, which may introduce clustering effects and influence the estimates of agreement. This should be considered when interpreting the results. A final limitation is that dynamic ultrasound and different tendon-loading conditions were not explored and may improve boundary detection in selected cases.

Future research should shift away from refining longitudinal measurements of the A1 pulley, which have shown limited reliability, and instead focus on pragmatic and reproducible sonographic reference landmarks that balance procedural safety with clinical effectiveness. In this context, the proximal border of the A2 pulley—shown here to have high anatomical consistency and strong inter-observer agreement—deserves further evaluation as a potential reference for guiding ultrasound-guided trigger finger release, particularly in non-cadaveric populations. From a clinical perspective, ultrasound should not be relied upon to define the exact longitudinal extent of the A1 pulley, but rather to identify pathological thickening and guide safe, function-oriented interventions.

## 5. Conclusions

High-resolution ultrasound showed better agreement with anatomical measurements for A2 pulley length compared to A1, and moderate reproducibility for non-pathological A1 thickness. However, its ability to define and measure the true anatomical length of the A1 pulley is inherently limited by anatomical variability, fiber discontinuity, and indistinct transitions toward A2. Therefore, A1 length should be interpreted cautiously in both clinical and interventional settings, whereas A1 thickness and A2-based anatomical references may represent more appropriate parameters to consider when evaluating flexor pulley pathology and guiding ultrasound-based procedures.

These findings challenge the assumption that A1 pulley length can be reliably measured using ultrasound and highlight the need to prioritize more reproducible parameters in clinical practice.

## Figures and Tables

**Table 1:** Descriptive measurements and paired comparisons of A1 and A2 pulleys.

Variable	O1 Mean ± SD (Range)	O2 Mean ± SD (Range)	O3 Mean ± SD (Range)	O1–O2 Mean ± SD (*p*)	O1–O3 Mean ± SD (*p*)	O2–O3 Mean ± SD (*p*)
A1 length (mm)	12.4 ± 2.7 (9.16–17.42)	9.6 ± 1.5 (7.48–12.93)	8.1 ± 2.1 (5–13)	2.80 ± 2.93 (<0.001)	4.31 ± 2.58 (<0.001)	1.51 ± 2.17 (0.006)
A2 length (mm)	16.3 ± 2.4 (11.5–20.6)	16.3 ± 2.7 (12.9–24.8)	15.6 ± 3.7 (10–25)	0.01 (0.98)	0.77 (0.16)	0.76 (0.18)
A1 thickness (mm)	0.41 ± 0.10 (0.26–0.68)	0.45 ± 0.10 (0.24–0.65)	—	−0.04 ± 0.08 (—)	—	—

**Table 2:** Reliability: ICC and Lin’s concordance.

Variable/Comparison	ICC (95% CI)	Lin’s CCC	Interpretation
A1 length O1 vs. O2	0.05 (−0.16–0.36)	0.05	Very poor
A1 length O1 vs. O3	0.16 (−0.09–0.49)	0.16	Poor
A1 length O2 vs. O3	0.22 (−0.13–0.56)	0.21	Poor
A2 length O1 vs. O2	0.83 (0.61–0.93)	0.82	Excellent
A2 length O1 vs. O3	0.71 (0.40–0.87)	~0.70	Good
A2 length O2 vs. O3	0.71 (0.42–0.87)	~0.70	Good
A1 thickness O1 vs. O2	0.61 (0.25–0.83)	0.60	Moderate

**Table 3:** Validity analysis: Bland–Altman and Passing-Bablok regression.

Variable	Comparison	Bias (mm)	Lower LoA	Upper LoA	Intercept (A)	95% CI A	Slope (B)	95% CI B
A1 length	O1 vs. O3	+4.31	−0.76	+9.38	9.11	3.78–13.30	0.44	−0.15–1.15
A1 length	O2 vs. O3	+1.51	−2.74	+5.76	7.41	5.29–11.10	0.24	−0.25–0.50
A2 length	O1 vs. O3	+0.77	−3.86	+5.40	8.58	5.03–11.41	0.52	0.33–0.77
A2 length	O2 vs. O3	+0.76	−3.99	+5.51	8.88	4.80–13.38	0.48	0.17–0.72

## Data Availability

The data presented in this study are available in the Supplementary Materials.

## Supplementary Materials

The following supporting information can be downloaded at: https://www.mdpi.com/article/10.3390/life16060867/s1.
