# Supplementary material for: Reliability and Anatomical Agreement of High-Resolution Ultrasound for Measuring the Length and Thickness of the A1 Pulley: A Cadaveric Study
Source: Life (Basel). 2026 May 22;16(6):867. doi: 10.3390/life16060867 (PMC13301609; doi:10.3390/life16060867)
Supplement: Supplementary file 1 [file life-16-00867-s001.zip › life-4229734-supplementary.pdf]

Finger_ ID	O1_A1_length (mm)	O1_A2_length (mm)	O1_A1_thickness (mm)	O2_A1_length (mm)	O2_A2_length (mm)	O2_A1_thickness (mm)	O3_A1_length (mm)	O3_A2_length (mm)
**1**	9,26	14,6	0,28	10,04	14,04	0,39	10	10
**2**	11,1	17,07	0,26	12,93	18,18	0,33	10	17
**3**	9,16	15,14	0,33	9,04	15,64	0,39	9	18
**4**	9,75	13,3	0,44	8,92	14,38	0,24	5	10
**5**	9,59	14,53	0,46	9,31	15,93	0,5	7	12
**6**	9,87	17,53	0,48	9,18	17,85	0,51	8	15
**7**	15,23	16,74	0,37	8,88	14,74	0,5	8	19
**8**	13,08	12,18	0,35	7,48	12,98	0,43	9	12
**9**	13,47	16,92	0,44	10,22	13,93	0,52	10	15
**10**	13,96	17,65	0,48	11,72	17,03	0,46	5	17
**11**	10,92	15,42	0,35	12,22	15,09	0,44	8	16
**12**	12,85	11,5	0,44	10,4	12,89	0,4	7	12
**13**	16,15	17,41	0,51	9,49	16	0,52	10	15
**14**	16,31	19,05	0,49	8,24	18,02	0,64	10	19
**15**	15,43	18,93	0,39	9,44	18,16	0,42	8	17
**16**	17,42	15,81	0,68	11,66	14,91	0,65	13	13
**17**	10,01	19,12	0,42	8,18	19,11	0,39	5	17
**18**	12,91	20,59	0,46	8,57	24,75	0,49	6	25
**19**	12,42	17,89	0,3	8,31	16,87	0,45	7	20
**20**	9,32	15,06	0,36	7,98	15,76	0,37	7	12
